# Primary cardiac sarcoma: insights from two decades of multimodal management at LMU Munich

**DOI:** 10.1186/s40959-025-00359-w

**Published:** 2025-06-26

**Authors:** Anton Burkhard-Meier, Dorit Di Gioia, Vindi Jurinovic, Michael Hoberger, Sinan E. Güler, Michael Völkl, Stefanie Corradini, Aurélie V. Gaasch, Annabel H. S. Alig, Thomas Knösel, Christian Hagl, Christian Schneider, Wulf Sienel, Wolfgang G. Kunz, Caspar Burkhard-Meier, Michael von Bergwelt-Baildon, Lars H. Lindner, Luc M. Berclaz

**Affiliations:** 1 Department of Medicine III, University Hospital, LMU Munich, Munich, Germany; 2 Bavarian Cancer Research Center (BZKF), Munich, Germany; 3 Institute for Medical Information Processing, Biometry, and Epidemiology, University Hospital, LMU Munich, Munich, Germany; 4 Department of Radiation Oncology, University Hospital, LMU Munich, Munich, Germany; 5 Institute of Pathology, LMU Munich, Munich, Germany; 6 Department of Cardiac Surgery, University Hospital, LMU Munich, Munich, Germany; 7 Department of Department of Thoracic Surgery, University Hospital, LMU Munich, Munich, Germany; 8 Department of Radiology, University Hospital, LMU Munich, Munich, Germany; 9 Kardiologische Gemeinschaftspraxis Viersen, Viersen, Germany; 10 German Cancer Consortium (DKTK), Partner Site Munich, Munich, Germany

**Keywords:** Primary cardiac sarcoma, Heart sarcoma, Soft tissue sarcoma, Multimodal treatment

## Abstract

**Background:**

Primary cardiac sarcomas (PCS) are rare, aggressive malignancies with poor prognosis and limited evidence guiding optimal management. We aimed to evaluate clinical and histopathological parameters in a single-center PCS cohort.

**Methods:**

Thirty-three patients diagnosed with PCS between 2002 and 2024 were retrospectively reviewed. Clinical outcomes and prognostic factors were analyzed. Event-free survival (EFS) was defined as the time from initial diagnosis to the first occurrence of disease progression, recurrence, or death. Overall survival (OS) was calculated from initial diagnosis to death.

**Results:**

The median age at diagnosis was 45 years, with angiosarcoma representing the most common histological subtype (*n* = 9, 27%). Most patients presented with localized or regional disease (*n* = 25, 75%), predominantly involving the right (*n* = 11, 33%) and left atrium (*n* = 8, 24%). In patients who underwent comprehensive genomic profiling, MDM2 amplification was the most common molecular alteration (*n* = 5, 45%). The majority of patients received multimodal treatment: surgical resection in 76% (*n* = 25), systemic therapy in 73% (*n* = 24), and radiotherapy in 21% (*n* = 7). After a median follow-up of 63.4 months, median EFS and OS were 11.7 months (95% CI 9.4–23.7) and 37.5 months (95% CI 21.2–83.2), respectively. Distant metastasis (*p* = 0.027, HR = 3.74) and angiosarcoma histology (*p* = 0.014, HR = 6.97) were significantly associated with worse OS, while surgical resection was associated with improved OS (*p* = 0.0064, HR = 0.086).

**Conclusion:**

Our findings underscore the key clinical and histopathological characteristics of PCS and suggest that surgical resection - even when incomplete - may confer a survival benefit in this aggressive tumor entity. The favorable clinical outcomes observed in this cohort may be attributable to the high proportion of patients undergoing multimodal treatment.

**Supplementary Information:**

The online version contains supplementary material available at 10.1186/s40959-025-00359-w.

## Introduction

Primary cardiac sarcomas (PCS) represent a rare and heterogeneous group of malignant tumors. While primary cardiac tumors are mostly benign, approximately 10% are malignant, and among these around 65% are PCS [1, 2]. The available literature primarily consists of case reports and limited case series. Compared to soft tissue sarcomas (STS) elsewhere, PCS skew toward younger patients (mean age ~ 45–50 years) [3]. Angiosarcoma appears to be the most common histological subtype in the heart, accounting for up to half of PCS cases [4, 5]. Other relatively frequent subtypes include undifferentiated pleomorphic sarcoma (UPS), leiomyosarcoma, and intimal sarcoma. The latter is characterized by MDM2 amplification, which may have been underdiagnosed in earlier studies due to the absence of comprehensive molecular profiling [6]. Overall, MDM2 amplification is considered the most common - and, with ongoing clinical trials, a potentially targetable - alteration in patients with PCS [7, 8]. Echocardiography remains the first-line diagnostic tool for cardiac tumors. However, computed tomography (CT) and cardiac magnetic resonance imaging (MRI) are increasingly utilized to enhance tissue characterization, determine exact tumor location, and aid in preoperative planning [9, 10]. Additionally, most patients undergo cardiac catheterization to assess coronary anatomy prior to surgery [11]. Optimal therapeutic management is challenging due to the lack of prospective trials. Given the tumor’s rarity and poor outcomes, therapy is often guided by principles from STS and individual institutional experience​. Surgical resection is the cornerstone whenever feasible, and the presence or absence of successful surgical resection is the strongest determinant of survival [12]. However, complete resection with negative margins (R0) is often difficult to achieve because of anatomic constraints and early metastasis [12]. Patients who undergo complete resection can occasionally achieve survival beyond 2–3 years: In a French Sarcoma Group report, patients with R0 resection had a median survival of ~ 38–39 months, versus ~ 18 months for those with residual disease (R1/R2 resection) and only ~ 11 months for those who could not be resected [12]. Additionally, multi-modality therapy appears to offer incremental benefits: Retrospective data suggest that combining surgery, chemotherapy, and radiation (when applicable) yields better survival than any single modality alone [9]. Stereotactic MR-guided radiotherapy (MRgRT) may offer a promising treatment alternative for inoperable cardiac tumor patients and is currently being investigated in a prospective multicenter trial initiated by our institution (DKRS00027108) [13, 14]. The optimal management of PCS is however still under debate, and prognostic factors remain unclear. Aim of this study was to analyze all PCS patients treated at a German high-volume sarcoma center regarding clinical and histopathological parameters.

## Materials and methods

### Study design

An explorative retrospective study design was chosen to address the research question. Patients with histologically confirmed PCS and initial diagnosis between February 2002 and October 2024 were included. The Internal Review Board and the Ethical Review Committee at the Ludwig-Maximilians-University (LMU) Hospital, Munich, Germany, approved the protocol of this research project (Protocol Nr. 25–0998).

### Patient selection and treatment

Eligible patients (age ≥ 18 years) had histologically proven STS or bone sarcoma with primary tumor localization in the heart including the pulmonary arteries. Clinical, pathological, and outcomes data were extracted from our prospectively maintained sarcoma database. Histopathologic diagnosis was reviewed by a specialized sarcoma pathologist. If available, results from comprehensive genomic profiling were evaluated. Patient follow-up was conducted with the assistance of the Bavarian Cancer Registry.

In general, surgery was performed in fit patients with localized or regional disease following comprehensive evaluation, including cardiac assessments. Anthracycline-based chemotherapy was administered to patients with high-risk localized/regional tumors (≥ 5 cm, G2-G3 according to Fédération Nationale des Centres de Lutte Contre le Cancer [FNCLCC]) and patients with metastatic disease. Radiotherapy was considered for patients with localized/regional disease who were not surgical candidates or in an adjuvant setting in selected cases.

CT imaging was performed with contrast enhancement. Staging was conducted using a Siemens SOMATOM Drive Dual Source CT scanner, covering the chest, abdomen, and pelvis in the portal-venous contrast phase with CAREkV for automatic tube voltage adaptation. CT reconstructions were performed with a 2 mm slice thickness for lung kernel and 3 mm for soft tissue kernel. All patients were examined in with a whole-body 1.5 Tesla MR scanner (MAGNETOM Aera, Siemens Healthineers). For the acquisition, electrocardiogram (ECG) and diaphragm triggers were used. Cardiac function was analyzed using ECG-gated imaging with the balanced steady state free precession (bSSFP) sequence as a stack in the short axis covering the whole heart in 8 to 10 slices and 1 slice in 2-chamber, 3-chamber, and 4-chamber views (SL 8 mm, TE 1.41ms, TR 30.6 ms). Late gadolinium enhancement was visualized with inversion recovery gradient echo pulse sequences after the administration of intravenous 0.15 mmol/kg bw Gadobutrol (Bayer Vital, Leverkusen, Germany). In selected cases, positron emission tomography with 18-fluorodeoxyglucose (FDG-PET-CT) was performed to further characterize metabolic tumor activity or to clarify ambiguous findings on conventional imaging. FDG uptake was quantified by calculating the maximal standardized uptake value (SUVmax), which indicates the degree of tumor metabolism.

### Statistical analysis

Event-free survival (EFS) and overall survival (OS) were analyzed using Cox proportional hazards regression. EFS was defined as the time from initial diagnosis to the first occurrence of disease progression, recurrence, or death. OS was calculated from initial diagnosis to death. Both EFS and OS were censored at the date of last follow-up. A p-value ≤ 0.05 was considered statistically significant. All statistical analyses were conducted using R software (version 4.0.3; R Foundation for Statistical Computing, Vienna, Austria).

## Results

### Patient characteristics

**Fig. 1:**
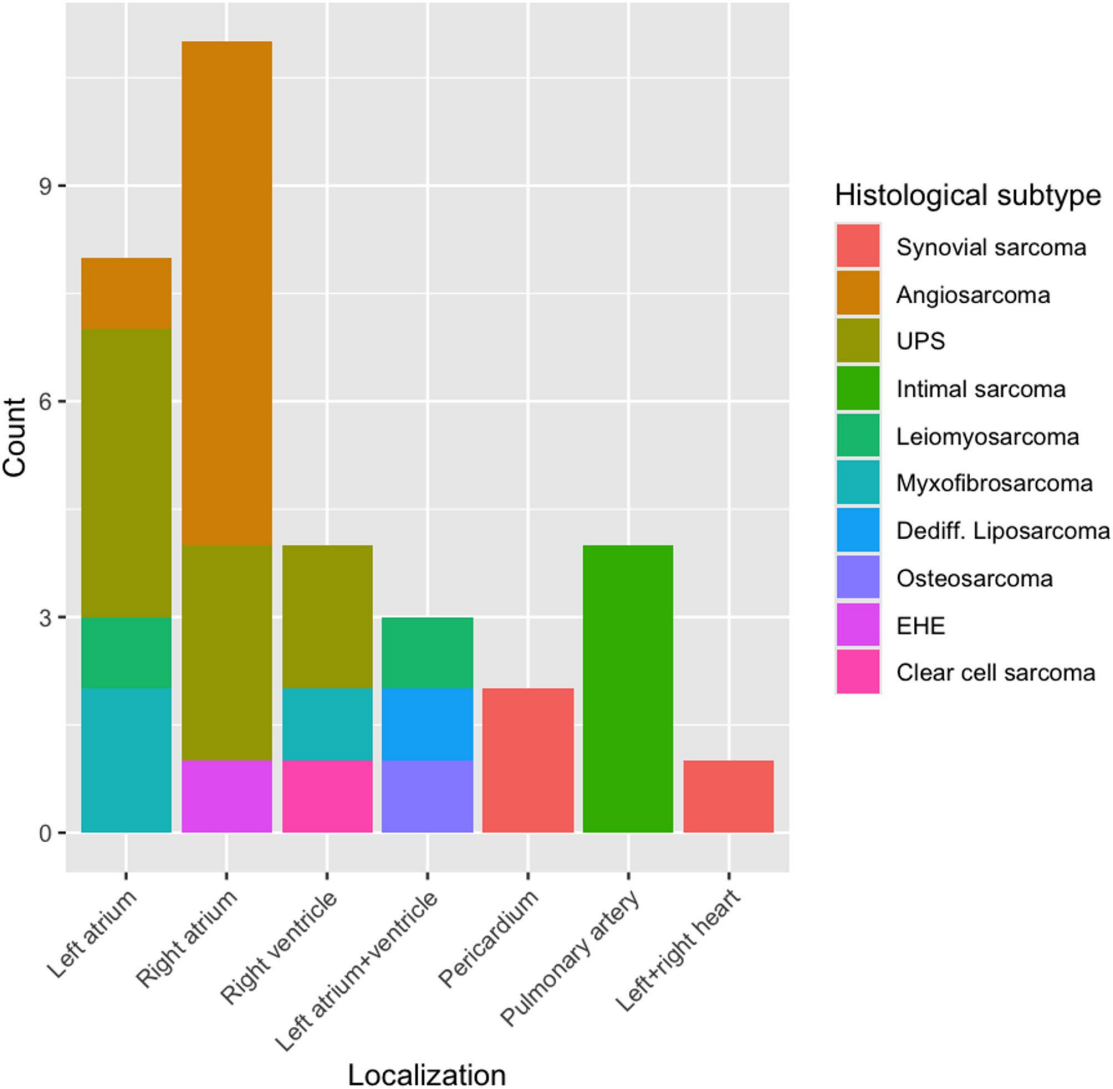
Histological subtypes with regard to PCS localization UPS: Undifferentiated pleomorphic sarcoma. EHE: Epithelioid hemangioendothelioma

The clinicopathologic characteristics of the patient cohort are summarized in Table 1. A total of 33 patients with PCS were included in this study. The median age at diagnosis was 45 years (range, 25–81 years), and 61% of patients (*n* = 20) were female. Angiosarcoma was the most frequent histological subtype (*n* = 9, 27%). The majority of tumors presented in a localized stage (*n* = 16, 48%), with the right atrium being the predominant site (*n* = 11, 33%). 78% of angiosarcoma were in the right atrium. The cardiac localization according to the different histological subtypes is shown in Fig. 1. Surgical resection was performed in 76% of cases (*n* = 25), while systemic therapy and radiotherapy were administered in 73% (*n* = 24) and 21% (*n* = 7) of patients, respectively. Information on symptoms at initial presentation was available for 79% of patients (*n* = 26). The most commonly reported symptoms included dyspnea (*n* = 17, 65%), decline in general condition (*n* = 8, 31%), and thoracic or abdominal pain (*n* = 3, 12%).

**Table 1:** Patient characteristics at baseline

Factor	Strata	*n*	%
Total		33	100
Age (years)	Median [Range]: 45 [25–81]		
Sex	Male Female	13 20	39 61
Histological subtype	Angiosarcoma Undifferentiated pleomorphic sarcoma Intimal sarcoma Myxofibrosarcoma Synovial sarcoma Leiomyosarcoma Clear cell sarcoma Dedifferentiated liposarcoma Epithelioid hemangioendothelioma Low-grade fibromyxoid sarcoma Osteosarcoma	9 7 5 2 3 2 1 1 1 1 1	27 21 15 6 9 6 3 3 3 3 3
Grading (FNCLCC)	G1 G2 G3 NA	3 6 22 2	9 18 67 6
Comprehensive genomic profiling	Yes No	11 22	33 67
Most common molecular alterations*	MDM2 amplification	5	45
	CDK4 amplification	2	18
	ERBB2 amplification	1	9
	ERBB3 amplification	1	9
	EWSR1::ATF1 fusion	1	9
	ACVR1 mutation	1	9
	PDGFRA amplification	1	9
	KIT amplification	1	9
	FGF10 amplification	1	9
	No alterations	3	27
Tumor stage	Localized Regional Distant metastasis	16 9 8	48 27 24
Primary tumor size (cm)	Median [Range]: 5.6 [2–18]		
Cardiac localization	Left atrium Right atrium Right ventricle Pulmonary artery Pericardium Extended over multiple sites	8 11 4 4 2 4	24 33 12 12 6 12
Pericardial effusion	Yes No Missing	10 21 2	64 30 6
(Pre-surgery) cardiac biopsy	Yes No	12 21	36 64
Left ventricular ejection fraction (LVEF)	Normal (≥ 55%) Mildly reduced (45–54%) Missing	23 4 6	70 12 18
Organ sites of synchronous metastasis*	Lung	2	25
	Lymph node	3	38
	Liver	3	38
	Bone	3	38
	Soft tissue	1	13
	Peritoneum	2	25
Organ sites of metastasis irrespective of timepoint*	Lung	12	36
	Lymph node	10	30
	Liver	7	21
	Bone	6	18
	Soft tissue	6	18
	Peritoneum	5	15
	Brain	2	6
FDG-PET-CT	Yes No	17 16	52 48
SUVmax	Median [Range]: 9.3 [5.5–18.3]		
Comorbidities	Coronary heart disease** Other cardiovascular condition only Others*** None Missing	5 5 4 16 3	15 15 12 48 9

*Multiple molecular alterations and organ sites of metastasis can be listed per patient. **In four patients, coronary heart disease was accompanied by an additional cardiovascular condition. ***Others include asthma, status post invasive breast cancer, and hypothyroidism.

FNCLCC: Fédération Nationale des Centres de Lutte Contre le Cancer. FDG-PET-CT: Fluorodeoxyglucose Positron Emission Tomography - Computed Tomography. SUVmax: Maximal standardized uptake value on FDG-PET-CT

### Molecular profiles

In 33% of patients (*n* = 11), comprehensive genomic profiling was conducted by next generation sequencing (NGS). The most common alterations were MDM2 amplification (*n* = 5, 45%) followed by CDK4 amplification (*n* = 2, 18%). In three patients, no alterations were detected. The histological subtypes with MDM2 amplification included three UPS and two intimal sarcomas. Additionally, two patients received a positive MDM2 fluorescence in situ hybridization (FISH) to confirm an intimal sarcoma diagnosis.

In one patient with metastatic low-grade myxofibrosarcoma and ERBB2 amplification along with positive HER2/neu immunohistochemistry, a targeted therapy with trastuzumab + pertuzumab was performed as a fifth-line treatment following heart transplantation. Given the patient’s exceedingly long disease stabilization (progression-free survival [PFS]: 14 months), additional patients with PCS were subsequently analyzed for HER2/neu expression. In a constructed tissue microarray with 15 evaluable patients, the patient with ERBB2 amplification was the only one with positive immunohistochemical HER2/neu expression.

### Treatment

Treatment modalities are summarized in Table 2. All patients received at least one form of therapy. As part of the primary treatment, surgical resection was performed in 76% of patients (*n* = 25), with complete (R0) resection achieved in 8% (*n* = 2). Systemic therapy was administered in 73% of cases (*n* = 24), and radiotherapy was applied in 21% (*n* = 7). In two cases, radiotherapy was applied in an adjuvant setting, while five patients were treated with stereotactic MRgRT. One patient underwent heart transplantation.

**Table 2:** Treatment modalities

Factor	Strata	*n*	%
Total		33	100
Number of treatment modalities	1 2 3	13 17 3	39 52 9
Resection	Yes No	25 8	76 24
Resection margins	R0 R1 R2	2 9 12	8 36 48
Radiotherapy	Yes No	7 26	21 79
Radiotherapy dose (Gy)	Median [Range]: 35 [35–50]		
Radiotherapy fractions	5 25	5 2	71 29
Systemic therapy	Yes No	24 9	73 27
First-line systemic therapy protocol	Doxorubicin + Ifosfamide Doxorubicin Doxorubicin + Olaratumab Doxorubicin + Ifosfamide + Cisplatin*	20 2 1 1	83 8 4 4
Best response to systemic therapy (RECIST)	Partial response (PR) Stable disease (SD) Progressive disease (PD) NA	10 8 1 4	43 35 4 17

*In accordance with the EURO-B.O.S.S. protocol [15]

RECIST: Response Evaluation Criteria In Solid Tumors

### Outcomes

At a median follow-up of 63.4 months, median EFS and OS from initial diagnosis were 11.7 months and 37.5 months, respectively. Early deaths or treatment discontinuations were rare in this cohort. In one patient with angiosarcoma histology, reduced cardiac function, and evidence of pulmonary and hepatic metastases, early death occurred within one month of initiating palliative chemotherapy. Treatment was not interrupted due to measured changes in left ventricular ejection fraction (LVEF). Survival outcomes were also assessed in specific subgroups with expected differences in clinical outcomes. In patients with localized/regional disease, the median EFS was 12.5 months and OS 48.2 months. In patients with metastatic disease, the median EFS was 7.2 months and OS 15.3 months. In patients with angiosarcoma (*n* = 9), median EFS was 9.8 months and OS 15.3 months. In patients with G1 histology (*n* = 3), median EFS was 18.8 months and OS 83.2 months. The patient with G1 myxofibrosarcoma who underwent heart transplantation achieved an EFS of 5.7 months and an OS of 66.0 months from the date of heart transplantation.

A uni- and multivariable analysis of clinical variables and their associations with EFS and OS is summarized in Table 3 and Supplementary Material (Table1) In univariate analysis, distant metastasis and angiosarcoma histology were associated with worse OS (*p* = 0.027, HR = 3.74 and 0.014, HR = 6.97, respectively) while resection was a positive prognostic factor for OS (*p* = 0.0064, HR = 0.086). In multivariate analysis, no significant associations were found. An additional univariate analysis restricted to patients with localized/regional disease is available in Supplementary Material (Table 2) In this analysis, cardiovascular comorbidities were associated with worse OS (*p* = 0.020, HR = 0.22).

**Table 3:** Univariate analysis of prognostic factors for event-free survival (EFS) and overall survival (OS)

		EFS		OS	
Factor	Strata	*p*-value	HR (95% CI)	*p*-value	HR (95% CI)
Age	≤ 45 vs. >45	0.26	0.63 (0.28–1.4)	0.24	0.56 (0.21–1.48)
Sex	Female vs. male	0.82	0.90 (0.38–2.14)	0.098	*0.39 (0.13–1.19)*
Disease stage	Regional vs. localized	0.59	0.77 (0.29–2.01)	0.87	0.91 (0.28–2.98)
	Distant vs. localized	0.32	1.65 (0.62–4.40)	**0.027**	**3.74 (1.16-12.0)**
Primary tumor localization	Right heart vs. left heart	0.42	0.69 (0.28–1.69)	0.74	1.20 (0.41–3.48)
	Pulmonary artery vs. left heart	0.69	0.76 (0.20–2.93)	*-*	*-*
	Others vs. left heart	0.57	0.57 (0.19–2.51)	0.82	1.16 (0.31–4.39)
Histological subtype	Angiosarcoma vs. others	*0.070*	*2.59 (0.92–7.23)*	**0.014**	**6.97 (1.48–32.74)**
Grading	G1 vs. G3	0.18	0.36 (0.08–1.59)	*0.083*	*0.16 (0.019–1.27)*
	G2 vs. G3	0.43	0.66 (0.24–1.82)	0.87	1.10 (0.36–3.39)
Primary tumor size (cm)	< 5.6 vs. ≥5.6	0.65	0.82 (0.35–1.92)	0.22	0.56 (0.22–1.43)
Pericardial effusion	Yes vs. No	0.90	0.95 (0.40–2.24)	0.31	1.66 (0.63–4.39)
LVEF	Normal vs. Mildly reduced	0.19	0.42 (0.12–1.53)	0.26	0.41 (0.085–1.94)
SUVmax	< 9.3 vs. ≥9.3	0.74	1.25 (0.33–4.74)	0.31	0.42 (0.079–2.22)
Resection	Yes vs. No	0.18	0.48 (0.17–1.40)	**0.0064**	**0.086 (0.015-0.50)**
Resection margins	R1/2 vs. R0	0.29	2.25 (0.49–10.2)	0.84	1.17 (0.25–5.43)
Systemic therapy	Yes vs. No	0.31	1.59 (0.65–3.85)	0.24	1.87 (0.66–5.34)
Best response to systemic therapy	PR vs. SD	0.55	1.41 (0.46–4.35)	0.49	1.57 (0.44–5.64)
Radiotherapy	Yes vs. No	0.88	1.08 (0.39–3.01)	0.57	1.46 (0.39–5.49)
Treatment modalities	1 vs. ≥2	0.62	0.81 (0.36–1.85)	0.84	1.10 (0.43–2.81)
Comorbidities	None/Other vs. Cardiovascular	0.87	0.93 (0.40–2.18)	*0.073*	*0.39 (0.14–1.09)*

LVEF: Left ventricular ejection fraction, SUVmax: Maximal standardized uptake value on FDG-PET-CT, PR: Partial response, SD: Stable disease

## Discussion

This study analyzed a single-center cohort of 33 patients with PCS treated between 2002 and 2024. Given the rarity and heterogeneity of PCS, detailed reporting of clinical presentation, treatment strategies, and outcomes is crucial to enhance the multidisciplinary management of affected patients.

In our cohort, the median patient age was 45 years which is in line with previous literature demonstrating that PCS is more likely to present in younger patients compared to other sarcoma subtypes [3, 5]. Angiosarcoma constitutes the most prevalent histological subtype of PCS and is linked to inferior outcomes relative to other subtypes, which was confirmed by our findings [12]. The cardiac localizations of histological subtypes were in line with previous literature. 24% presented with synchronous metastases and 73% developed metastatic disease during the course of the disease. The uncommon cases of lymph node metastasis in this cohort were strongly related to the high proportion of angiosarcoma [16]. Two patients developed brain metastasis during the course of disease, which is in line with a previously published study demonstrating a high propensity for brain metastasis in patients with PCS [17].

Due to young patient age and limited therapeutic options, comprehensive genomic profiling was performed in one third of cases as part of routine diagnostics. MDM2 amplifications emerged as the most frequent molecular alteration, consistent with findings in patients with intimal sarcoma and cardiac UPS [6, 18]. Although some studies suggest that these two subtypes may represent a single entity, the current WHO classification differentiates them based on tumor location [19]. MDM2 inhibitors such as brigimadlin have been evaluated in recent clinical trials and may offer a promising targeted treatment approach for patients with PCS [7, 8]. Following the observed clinical benefit in one patient with ERBB2 amplification treated with trastuzumab + pertuzumab, additional patients were analyzed for HER2/neu expression. HER2/neu could not be identified as a therapeutic target in any of the other analyzed patients. However, given the emergence of targeted therapies and the identification of potentially actionable alterations in the majority of evaluated patients, comprehensive genomic profiling should be routinely implemented in PCS, similar to other advanced sarcomas as highlighted in a previous analysis from our institution [15].

At a median follow-up of 63.4 months, the median OS from first diagnosis was 37.5 months which compares favorably to previously reported cohorts with survival ranging from 6 to 25 months [4, 5, 9, 12, 17, 20]. Although not statistically significant in univariate analysis, this may reflect the multimodal treatment in our cohort with 61% of patients receiving at least two treatment modalities as part of the primary therapy. 83% of patients with systemic therapy received a combination of doxorubicin + ifosfamide (AI). Judson et al. demonstrated a PFS and objective response rate (ORR) but no OS benefit for AI vs. doxorubicin monotherapy in locally advanced and metastatic STS patients [21]. These findings resulted in doxorubicin monotherapy as the standard treatment for inoperable STS patients with the exception of leiomyosarcoma, where more recent studies indicate a survival benefit with the combination of doxorubicin and dacarbazine or trabectedin [22–24]. The relatively long survival rates in our cohort may indicate the potential benefit of AI in PCS where tumor shrinkage could be particularly relevant. Overall, our findings confirm that surgical resection remains the most important component of treatment in PCS. Resection was significantly associated with an OS benefit despite of only very few patients (8%) achieving complete resection. The low rate of R0 resections highlights the need for additional or alternative treatment modalities including feasible radiotherapy protocols such as stereotactic MRgRT, which is currently under evaluation in a prospective clinical trial (DRKS00027108).

In our cohort, 30% had a cardiovascular comorbidity. As PCS mainly affects younger patients (median age in our cohort: 45 years), this proportion of cardiac comorbidities was high compared to the prevalence of cardiovascular disease in the general population and reflects the need for diagnostic workup prior to multimodal treatment such as anthracycline-based chemotherapy or surgery [25]. In patients with localized/regional disease, the presence of cardiovascular comorbidities was associated with worse OS. Given the localization of the tumors and the intensive cardiotoxic treatment required, cardiovascular comorbidities may pose a more significant risk factor in PCS than in other STS.

One patient with low-grade (G1) cardiac myxofibrosarcoma underwent heart transplantation and achieved an OS of 66 months, which is notably favorable compared to published outcomes for PCS, even in a low-grade setting. In addition to the performed transplantation, the prolonged survival may in part be attributed to a sustained response to adjuvant therapy with trastuzumab + pertuzumab in the setting of strong Her2/neu expression. While the role of heart transplantation in PCS remains controversial, it may offer a survival benefit in select patients with low-grade tumors and more favorable histologic subtypes, as also suggested by Li and colleagues [26].

Our study is subject to several limitations, most notably the heterogeneity of the cohort, typical in the setting of ultra-rare tumors. Survival analyses were only feasible in select subgroups, such as patients with localized/regional disease and angiosarcoma histology, and the small sample size did not allow a robust multivariate analysis of prognostic factors. Additionally, the retrospective nature of the study limited the availability of detailed information such as clinical symptoms at the time of initial diagnosis or long-term follow-up of cardiac function after anthracycline-based chemotherapy. Lastly, due to the inclusion of patients between 2002 and 2024, imaging modalities evolved over time, which leads to a variability regarding potential early detection and radiologic tumor characterization. Prospective studies in patients with PCS are urgently needed. Until such data become available, single-center analyses like ours can offer valuable insights and help inform treatment strategies for this rare and challenging disease.

## Electronic supplementary material

Below is the link to the electronic supplementary material.

Supplementary Material

## Data Availability

The data that support the findings of this study are not openly available due to reasons of sensitivity and are available from the corresponding author upon reasonable request.

## Abbreviations

LMU: Ludwig-Maximilians-University Munich
PCS: Primary Cardiac Sarcoma
STS: Soft Tissue Sarcoma
UPS: Undifferentiated Pleomorphic Sarcoma
CT: Computed Tomography
MRI: Magnetic Resonance Imaging
MRgRT: MR-guided Radiotherapy
EFS: Event-Free Survival
OS: Overall Survival
FNCLCC: Fédération Nationale des Centres de Lutte Contre le Cancer
ECG: Electrocardiogram
bSSFP: balanced Steady State Free Precession
LVEF: Left Ventricular Ejection Fraction
FDG-PET-CT: Fluorodeoxyglucose Positron Emission Tomography - Computed Tomography
SUVmax: Maximal standardized uptake value on FDG-PET-CT
FISH: Fluorescence In Situ Hybridization
RECIST: Response Evaluation Criteria In Solid Tumors
PR: Partial Response
SD: Stable Disease
PD: Progressive Disease
AI: Doxorubicin + Ifosfamide
ORR: Objective Response Rate
