# Supplementary material for: Primary cardiac sarcoma: insights from two decades of multimodal management at LMU Munich
Source: Cardiooncology. 2025 Jun 26;11:58. doi: 10.1186/s40959-025-00359-w (PMC12199525; doi:10.1186/s40959-025-00359-w)
Supplement: Supplementary file 1 — Supplementary Material [file 40959_2025_359_MOESM1_ESM.docx]

Supplementary Material

Table 1: Multivariate analysis of prognostic factors for event-free survival (EFS) and overall survival (OS)

|  |  | **EFS** | | **OS** | |
| --- | --- | --- | --- | --- | --- |
| **Factor** | **Strata** | **p-value** | **HR (95% CI)** | **p-value** | **HR (95% CI)** |
| Disease stage | Metastatic vs. Regional/Localized | 0.43 | 1.78 (0.43-7.39) | 0.55 | 1.62 (0.34-7.85) |
| Histological subtype | Angiosarcoma vs. Others | 0.20 | 2.10 (0.67-6.61) | 0.11 | 4.01 (0.74-21.80) |
| Grading | G2/3 vs. G1 | 0.50 | 1.73 (0.35-8.71) | - | - |
| Resection | Yes vs. No | 0.96 | 1.04 (0.21-5.08) | 0.13 | 0.19 (0.023-1.58) |
| Radiotherapy | Yes vs. No | 0.81 | 1.15 (0.37-3.58) | **-** | **-** |
| Systemic therapy | Yes vs. No | 0.59 | 1.39 (0.42-4.55) | - | - |

Table 2: Univariate analysis of prognostic factors for event-free survival (EFS) and overall survival (OS) in patients with localized/regional disease

|  |  | **EFS** | | **OS** | |
| --- | --- | --- | --- | --- | --- |
| **Factor** | **Strata** | **p-value** | **HR (95% CI)** | **p-value** | **HR (95% CI)** |
| Age | ≤45 vs. >45 | *0.075* | *0.42 (0.16-1.09)* | 0.20 | 0.47 (0.15-1.5) |
| Sex | Female vs. male | 0.71 | 1.22 (0.43-3.41) | 0.13 | 0.35 (0.09-1.34) |
| Primary tumor localization | Right heart vs. left heart | 0.27 | 0.55 (0.19-1-56) | 0.69 | 1.30 (0.36-4.75) |
|  | Pulmonary artery vs. left heart | 0.38 | 0.47 (0.087-2.54) | *-* | *-* |
|  | Others vs. left heart | 0.52 | 0.64 (0.17-2.45) | 0.51 | 1.62 (0.39-6.74) |
| Histological subtype | Angiosarcoma vs. Others | 0.33 | 1.95 (0.51-7.46) | 0.29 | 3.76 (0.32-44.09) |
| Grading | G1 vs. G3 | 0.26 | 0.42 (0.092-1.89) | 0.12 | 0.18 (0.022-1.53) |
|  | G2 vs. G3 | 0.80 | 0.85 (0.24-3.04) | 0.77 | 1.24 (0.30-5.11) |
| Primary tumor size (cm) | <5.6 vs. ≥5.6 | 0.59 | 0.75 (0.26-2.16) | 0.16 | 0.45 (0.15-1.36) |
| Pericardial effusion | Yes vs. No | 0.37 | 0.62 (0.22-1.77) | 0.80 | 1.17 (0.35-3.93) |
| LVEF | Normal vs. Mildly reduced | 0.22 | 0.37 (0.077-1.82) | 0.51 | 0.49 (0.057-4.19) |
| SUVmax | <9.3 vs. ≥9.3 | 0.82 | 1.20 (0.26-5.43) | 0.21 | 0.24 (0.026-2.21) |
| Resection | Yes vs. No | 0.85 | 1.21 (0.16-9.35) | *0.075* | *0.079 (0.0048-1.30)* |
| Resection margins | R1/2 vs. R0 | 0.16 | 3.0 (0.64-14.1) | 0.94 | 1.06 (0.22-5.08) |
| Systemic therapy | Yes vs. No | 0.84 | 1.11 (0.42-2.92) | 0.44 | 1.59 (0.49-5.2) |
| Best response to systemic therapy | PR vs. SD | 0.73 | 1.26 (0.33-4.76) | 0.89 | 0.90 (0.20-4.05) |
| Radiotherapy | Yes vs. No | 0.97 | 1.02 (0.32-3.29) | 0.17 | 2.76 (0.64-11.85) |
| Treatment modalities | 1 vs. ≥2 | 0.65 | 0.79 (0.28-2.21) | 0.54 | 0.69 (0.21-2.25) |
| Comorbidities | None/Other vs. Cardiovascular | 0.34 | 0.59 (0.20-1.74) | **0.020** | **0.22 (0.060-0.78)** |
